# Income Support Needs and Bedside Legal Assistance for Patients Recovering From Violent Injuries

**DOI:** 10.1001/jamanetworkopen.2025.38044

**Published:** 2025-10-16

**Authors:** Elizabeth L. Tung, Rhea Pillai, Nisha Sen-Gupta, Alexander Nigro, Franklin Cosey-Gay, Bradley C. Stolbach, Selwyn O. Rogers, Tanya L. Zakrison

**Affiliations:** 1 Section of General Internal Medicine, Department of Medicine, University of Chicago, Chicago, Illinois; 2 Center for Health and the Social Sciences, University of Chicago, Chicago, Illinois; 3 Urban Health Initiative, University of Chicago, Chicago, Illinois; 4 Section of Developmental and Behavioral Pediatrics, Department of Pediatrics, University of Chicago, Chicago, Illinois; 5 Section of Trauma & Acute Care Surgery, Department of Surgery, University of Chicago, Chicago, Illinois

## Abstract

**Importance:**

Economic exclusion is a structural disadvantage that is challenging to address in violence reduction and recovery programs. Medical-legal partnership (MLP) is an effective practice for addressing poverty and increasing access to income support and other public benefits programs.

**Objective:**

To examine the legal needs and access to public benefits among patients enrolled in Recovery Legal Care, a bedside MLP that supports patients recovering from violent injuries.

**Design, Setting, and Participants:**

This cohort study involved patients recovering from violent injuries at an academic level 1 trauma center in Chicago, Illinois, who were enrolled in the Recovery Legal Care program and underwent legal needs screening and assessment from November 16, 2022, to November 11, 2024, with follow-up of financial benefits through June 30, 2025.

**Main Outcomes and Measures:**

The main outcome were the number and types of legal needs reported by patients, as well as the financial benefit received. Legal needs were screened using the I-HELP (Income, Housing & Utilities, Education & Employment, Legal Status, and Personal & Family Stability) tool, followed by an 18-item assessment of specific public benefits needs; financial benefit was measured as a total lump sum financial benefit and annualized financial benefit.

**Results:**

A total of 516 participants (median [IQR] age, 32 [24-40] years) were screened for legal needs; 437 (84.7%) were men; 439 (85.1%) self-identified as Black, non-Hispanic; 56 (10.9%) as Hispanic, Latino, Latina, or Latinx; 12 (2.3%) as White, non-Hispanic; and 3 (0.6%) as other race and ethnicity (American Indian and Alaska Native, Asian, and Native Hawaiian or Other Pacific Islander). Among participants screened, 489 (94.8%) had at least 1 legal need, with income (461 [89.3%]), housing (332 [64.3%]), and employment (307 [59.5%]) being the most frequent needs. Most participants (406 [88.1%]) reported needing assistance with at least 1 of the listed benefit types, with 115 (25.0%) reporting barriers to accessing benefits in the past (eg, denial). Need for assistance was associated with living in the highest quartile of neighborhood disadvantage (adjusted odds ratio, 5.68; 95% CI, 1.05-30.71) relative to the lowest. During the study period, 694 legal cases were opened among 457 participants referred for legal intake. Common cases involved needs related to public benefits, criminal records expungement, employment, and housing. Of the 409 (58.9%) cases closed, 134 (32.8%) involved direct legal representation with financial benefit, yielding a total lump sum financial benefit of $264 068.10 and annualized financial benefit of $482 997.60 in recurring payments to patients.

**Conclusions and Relevance:**

In this cohort study, patients recovering from violent injuries had nearly universal legal needs, particularly for income support with public benefits. Legal assistance may be a pragmatic solution, infrequently leveraged in addressing violence, that can increase access to income support and other public benefits programs.

## Key Points

**Question:**

Do patients recovering from violent injuries have income support needs, and is bedside legal assistance associated with financial benefit?

**Findings:**

In this cohort study of 516 patients admitted with violent injuries at an urban trauma center, 94.8% had at least 1 legal need, with income support (required by 89.3% of participants) being the most frequent need. The financial benefits of bedside legal assistance included $264 068 in lump sum payments and $482 998 in recurring payments to patients over 2 years.

**Meaning:**

This study found that legal needs among patients recovering from violent injuries are nearly universal, particularly the need for income support with public benefits; bedside legal assistance may be one solution for addressing needs.

## Teaser

This cohort study assesses the legal needs and access to public benefits of patients enrolled in a medical-legal partnership that assists individuals recovering from violent injuries.

## Introduction

Violence reduction and recovery programs often have difficulty addressing root causes, particularly the systematic denial of economic opportunities to racial and ethnic minority groups. This economic exclusion was codified via Jim Crow–era policies that deliberately excluded Black workers from public benefits, such as Social Security, labor protections, and employer-based health insurance.^1,2,3,4^ Discriminatory lending and restricted access to New Deal programs further concentrated poverty in Black communities, with concomitant proliferation of community violence.^5,6^ In Chicago, Illinois, the 15 most segregated and disinvested community areas—comprising only 19% of the city overall—account for over 50% of shootings.^7^ Correspondingly, a cohort study of 2311 children in Baltimore found that childhood neighborhood poverty, shaped by these historical practices, was one of the only factors consistently associated with violent death by mid adulthood.^8^

While public benefits exist to help mitigate poverty and economic hardship, growing literature has documented unequal and inconsistent administration of public benefits programs. In a study of income support programs, only 1 in 5 eligible families received assistance from programs they were entitled to, including the Earned Income Tax Credit and Temporary Assistance for Needy Families (TANF).^9^ Black recipients were more likely to face benefit-related sanctions (eg, discontinuation) at the discretion of caseworkers. In another study of TANF policies, families who experienced barriers to TANF access had higher rates of child neglect and foster care placements.^10^ Other studies have found similar associations for the Supplemental Nutrition Assistance Program (SNAP), which supports food security for needy families. In one study, access to SNAP was associated with reductions in Child Protective Services involvement.^11^ There is substantial evidence that racial inequities in access to public benefits can have adverse effects on denied children and families, including increased risk for violence.

Legal assistance programs can address the structural disadvantages that impact poverty and access to public benefits programs. Medical-legal partnership (MLP) is a model that has been increasingly adopted as a powerful tool to address upstream and structural factors.^12^ MLPs embed legal experts into health care teams to address health-harming legal needs (HHLNs), a special category of social determinants of health that can be addressed with civil legal remedies.^13^ For example, MLPs can advocate on behalf of individuals and families to address unlawfully denied public benefits (eg, food assistance and childcare assistance), inequitable housing opportunities, or unenforced environmental regulations.

These programs have proliferated in the past decade, in part, due to recognition that populations with historical exclusion from benefits programs often require legal advocacy to resolve their needs. For families affected by violent injury, who are at uniquely high risk of adverse involvement with the justice system, this type of legal advocacy is often critical for overcoming challenges to accessing income support and other benefits. In turn, vulnerability to social, economic, and legal exclusion can cyclically exacerbate community violence. Thus, MLPs may be particularly impactful for communities affected by violence by addressing barriers to recovery as well as upstream social and economic factors that increase the risk for violence in the first place. Berkowitz and Palakshappa^14^ noted that “in the absence of a clear account of how poverty predictably emerges from a society’s distributive institutions, people may turn to accounts that emphasize the behavioral deficiencies or cultural inferiority of those who experience poverty,” rather than the structural or root causes of poverty and its consequences.

Recovery Legal Care is an MLP that offers bedside legal assistance to support the social and economic needs of survivors of violence. Despite substantial evidence of economic hardship in communities affected by violence, few studies have quantified the income support needs of this population. Even fewer studies have specifically reported on the levels of access or lack thereof to the public benefits economy—potentially a structural lever of economic exclusion. The purpose of this study is to examine and quantify HHLNs, with an emphasis on the need for income support, among patients with violent injuries. We hypothesized high rates of need exist for income support along with a corresponding need for assistance in accessing the public benefits economy. We also examined the geographic distribution of public benefits access and financial benefits received via legal assistance during the 2-year study period.

## Methods

### Setting and Design

In this retrospective cohort study involving patients recovering from violent injuries who participated in the Recovery Legal Care program, we analyzed data collected during routine legal needs screening and assessment (November 16, 2022, to November 11, 2024), with follow-up of financial benefits through June 30, 2025. The trauma center’s community service area is on the South Side of Chicago, an urban area that encompasses 12 zip codes and a population that primarily identifies as Black (73.7%) or Hispanic, Latino, Latina, or Latinx (14.6%).^15^ The area’s economic hardship index is 77.8, which is 25.1% higher than the overall city.^15^ This study was approved by the University of Chicago Institutional Review Board with a waiver of informed consent due to retrospective analysis of deidentified data. We followed the Strengthening the Reporting of Observational Studies in Epidemiology (STROBE) reporting guideline.

Research staff approached patients who were violently injured and admitted to the inpatient trauma service. Due to a limited number of program openings each week, a random number generator (1 to n) was used to determine the order of recruitment; patients were offered program services until the legal team was at capacity. Inclusion criteria were (1) treatment for an intentional interpersonal violent injury; (2) age 14 years or older; (3) able to provide informed consent or assent; and (4) residing at an Illinois address. For participants 14 to 17 years old, consent was obtained from 1 parent or legal guardian. Violent injuries included domestic or nondomestic firearm injuries, other penetrating injuries (eg, stab wounds), and nonpenetrating or blunt injuries (eg, physical fighting, sexual assault). Exclusion criteria were (1) diagnosis of severe mental illness (ie, psychosis, suicidality); (2) unable to participate due to mental status; (3) prior receipt of legal services within 1 year; and (4) currently incarcerated.

### Legal Needs Screening and Assessment Data

For eligible patients, research staff completed legal needs screening and assessment. The legal needs screening consisted of 5 items adapted from the I-HELP (Income, Housing & Utilities, Education & Employment, Legal Status, and Personal & Family Stability) tool developed by the National Center for Medical-Legal Partnership to describe common HHLNs.^12^ Each screen with a positive result was followed by a more in-depth assessment of specific needs. For example, a screen that was positive for HHLNs related to income (a yes response to the question “currently or in the past month, have you been concerned about having money to pay for your basic expenses?”) was followed by an 18-item assessment. The assessment included queries about 10 common benefit types: (1) Medicare or Medicaid, (2) disability, (3) cash assistance, (4) childcare assistance, (5) SNAP benefits, (6) unemployment, (7) child support, (8) retirement, (9) Women, Infants, and Children benefits, and (10) utility assistance. Participants were also asked about their experiences with each benefit type: (1) receipt of benefit, (2) barriers or issues with benefit, and (3) need for assistance with benefit.

### Geographic Data

Participant addresses were geocoded to Census block groups and paired to the 2022 Area Deprivation Index (ADI) rankings.^16^ The ADI is a measure of socioeconomic disadvantage for a neighborhood, with a neighborhood defined as a Census block group. The measure is calculated as a national percentile ranking with 100 as the highest level of disadvantage using socioeconomic factors from the 2018 to 2022 American Community Survey 5-Year Estimates. Factors included in its derivation are income and poverty, educational attainment, employment and labor, household composition, housing characteristics, and basic material resources.^16^

### Financial Benefits Data

Financial benefits data were obtained from databases maintained by our legal partner (Legal Aid Chicago), which included detailed information about each case opened. Measures of financial benefit included: (1) total annualized financial benefit, defined as the dollar sum of monthly financial benefits to all participants over 1 year; (2) mean monthly benefit, defined as the dollar mean of all monthly benefits across each benefit category; and (3) mean lump sum benefit, defined as the dollar mean of all lump sum benefits across each benefit category. Common monthly benefits included monthly Supplemental Security Income (SSI) or cash assistance payments; common 1-time lump sum benefits included back payment for previously denied benefits, debt forgiveness, or Crime Victims Compensation (CVC).

### Participants’ Race, Ethnicity, and Gender

Race and ethnicity data were self-reported by participants and were included to assess for potential inequities. Race and ethnicity categories in this study were combined by the investigators into the following categories: Black, non-Hispanic (hereafter Black); Hispanic or Latino, Latina, or Latinx; White, non-Hispanic (hereafter White); other; or declined or unknown. Other included American Indian or Alaska Native, Asian, Native Hawaiian or Pacific Islander, and other responses. Participants selected from investigator-defined gender terms, including woman, man, nonbinary, transgender woman, transgender man, or other. The other gender category included a free-text option to specify other gender, but this category was not selected by any participants.

### Statistical Analysis

Participant characteristics were described for the overall sample. Descriptive statistics were calculated to quantify the prevalence of HHLNs in our cohort, participants’ experiences with each benefit type, and financial benefits received.

Mixed-effects logistic regression models, with a random intercept for Census block group, were used to examine participants’ need for assistance as a function of individual demographic characteristics and neighborhood disadvantage (measured using ADI). All analyses clustered participants within their Census block group of residence and adjusted for age, gender, race and ethnicity, preferred language, insurance type, and injury type. Analyses were performed using Stata SE, version 17 (StataCorp). Two-sided *P* < .05 was considered statistically significant.

## Results

During the study period, 606 patients with violent injuries were eligible and approached for participation in the Recovery Legal Care program, of whom 516 (85.1%) agreed to be screened for legal needs (Table 1). Among participants who were screened, 437 were men (84.7%) and 79 were women (15.3%); 439 (85.1%) self-identified as Black, 56 (10.9%) as Hispanic, Latino, Latina, or Latinx, 12 (2.3%) as White, and 3 (0.6%) as other race and ethnicity. Participants had a median (IQR) age of 32 (24-40) years. In all, 294 participants (57.0%) were insured by Medicaid or had dual Medicare-Medicaid eligibility, while 160 (31.0%) had no or unknown insurance at the time of admission. Most participants (367 [71.1%]) were admitted for firearm injury.

**Table 1.** Characteristics of Recovery Legal Care Participants

Characteristic	No. (%) (N = 516)^a^^,^^b^
Age, y	
<18	37 (7.2)
18-26	132 (25.6)
27-34	141 (27.3)
≥35	206 (39.9)
Gender^c^	
Woman	79 (15.3)
Man	437 (84.7)
Racial and ethnicity	
Black	439 (85.1)
Hispanic or Latino, Latina, or Latinx	56 (10.9)
White	12 (2.3)
Other^d^	3 (0.6)
Declined or unknown	6 (1.2)
Preferred language	
English	468 (90.7)
Spanish	21 (4.1)
Other^e^	2 (0.4)
Declined or unknown	25 (4.8)
Insurance status	
Medicaid or dual-eligible	294 (57.0)
Medicare only	4 (0.8)
Commercial	57 (11.1)
Other governmental^f^	1 (0.2)
Uninsured or unknown	160 (31.0)
Mechanism of violent injury	
Firearm injury	367 (71.1)
Other penetrating injury	59 (11.4)
Nonpenetrating or blunt injury	90 (17.4)

^a^ Total includes all participants who were screened for legal needs, including those with negative screening (n = 27).

^b^ Percentages may not equal 100 due to rounding.

^c^ Additional gender categories (nonbinary, transgender woman, transgender man, or other) were provided to participants as options but were not selected.

^d^ Other includes American Indian and Alaska Native (n = 1), Asian (n = 1), and Native Hawaiian or Other Pacific Islander (n = 1).

^e^ Other included French and Portuguese.

^f^ Other governmental insurance included Veterans Health Insurance.

Overall, 489 participants (94.8%) had a positive screen for at least 1 HHLN (Table 2) and were referred to our legal team for intake. Most participants (461 [89.3%]) had a positive screen for HHLNs related to income, 332 (64.3%) had a positive screen for HHLNs related to housing and utilities, and 307 (59.5%) had positive screening for employment and education. HHLNs related to personal and family stability, such as orders of protection, child custody issues, or criminal records expungement, were reported in 238 participants (46.1%). A smaller number of participants reported HHLNs related to legal status and immigration (21 [4.1%]).

**Table 2.** Legal Needs of Recovery Legal Care Participants

I-HELP category^a^	Participants with a screen positive for legal needs, No. (%) (N = 516)
Income	461 (89.3)
Housing and utilities	332 (64.3)
Employment and education	307 (59.5)
Legal status and immigration	21 (4.1)
Personal and family stability	238 (46.1)
Any legal need	489 (94.8)

Abbreviation: I-HELP, Income, Housing & Utilities, Education & Employment, Legal Status, and Personal & Family Stability.

^a^ The I-HELP screening tool was developed by the National Center for Medical-Legal Partnership to describe common health-related social and legal needs.^13^

### Access to the Public Benefits Economy

We conducted a detailed assessment of engagement and need for assistance with the public benefits economy in the 461 participants with a positive screen for HHLNs related to income (Table 3). Of the public benefits assessed, Medicaid (n = 252 [54.7%]) and SNAP (n = 268 [58.1%]) were the most frequently received benefits. Only 82 participants (17.8%) reported receiving any other public benefit. The largest gaps in received compared with needed public benefits were among participants eligible for Aid to the Aged, Blind, and Disabled cash assistance (received, 5 [1.1%]; needed, 144 [31.2%]) and disability support (received, 55 [11.9%]; needed, 330 [71.6%]). One hundred and fifteen participants (25.0%) reported a barrier or issue with a benefit in the past, with 67 (14.5%) reporting barriers related to SNAP alone. Most participants (406 [88.1%]) reported needing assistance with at least 1 of the public benefit types assessed.

**Table 3.** Public Benefit Categories of Recovery Legal Care Participants

Category	Participants, No. (%) (n = 461)^a^		
	Currently receives the benefit	Has had a barrier or issue with the benefit	Desires or needs assistance with the benefit
Medicaid	252 (54.7)	34 (7.4)	99 (21.5)
Social Security Disability Insurance or Supplemental Security Income	55 (11.9)	25 (5.4)	330 (71.6)
Aid to the Aged, Blind, and Disabled	5 (1.1)	4 (0.9)	144 (31.2)
Temporary Assistance for Needy Families	3 (0.7)	4 (0.9)	75 (16.3)
Supplemental Nutrition Assistance Program	268 (58.1)	67 (14.5)	173 (37.5)
Child support	25 (5.4)	10 (2.2)	15 (3.3)
Any benefit	345 (74.8)	115 (25.0)	406 (88.1)

^a^ Public benefits items were queried among the 461 participants with a positive screen for income needs.

Overall, 418 (90.7%) of the 461 participants with a need related to income provided a valid address that could be geocoded, with an additional 25 (5.4%) reporting they were unhoused, 9 (2.0%) reporting invalid addresses, and 9 (2.0%) with missing addresses. Among participants with a valid address, the median (IQR) ADI was 74 (60-86). Most participants (369 [88.3%]) lived in a neighborhood with high disadvantage (ADI, 51-100), and half (213 [50.9%]) lived in a neighborhood in the highest quartile of disadvantage (ADI, 76-100). Participants living in neighborhoods with higher disadvantage had similar rates of receiving public benefits compared with those with lower disadvantage (68.7% vs 72.3%; *P* = .61) (eTable in Supplement 1). However, participants in the highest quartile of disadvantage had higher adjusted odds (adjusted odds ratio, 5.68; 95% CI, 1.05-30.71) of needing assistance with at least 1 public benefit type compared with those in the lowest quartile (Table 4).

**Table 4.** Association Between Area Deprivation Index and Need for Assistance With Public Benefits

ADI quartile	Need for assistance with public benefits^a^		
	No./total No. (%) (n = 418)	OR (95% CI)	AOR (95% CI)
Lowest	4/7 (57.1)	1 [Reference]	1 [Reference]
Medium	32/40 (80.0)	3.00 (0.56-16.19)	3.40 (0.54-21.50)
High	135/173 (78.0)	2.66 (0.57-12.42)	3.28 (0.61-17.60)
Highest	171/198 (86.4)	4.75 (1.01-22.40)	5.68 (1.05-30.71)

Abbreviations: ADI, Area Deprivation Index; AOR, adjusted odds ratio; OR, odds ratio.

^a^ Mixed effects logistic regression models were used to cluster participants within the 325 Census block groups represented in this study.

### Financial Benefits

During the 2-year study period, 457 participants were referred to our legal team for intake. Of those with a screen positive for HHLNs, 32 participants were excluded due to various reasons, including legal residence outside of Illinois, placement into police custody, or incomplete authorization forms; however, their demographic characteristics were similar to the overall sample, with the majority being men (25 [78.1%]) and of Black race (25 [78.1%]), with a median (IQR) age of 30 (21-40) years. In total, 694 legal cases were opened (mean [SD], 1.5 [0.6] cases per patient). Common legal cases involved the acquisition of public benefits, criminal records relief, employment needs, and housing needs. To date, 409 legal cases (58.9%) have been closed, of which 134 (32.8%) involved direct legal representation for public benefits with a financial outcome (Table 5). In total, cases yielded a lump sum financial benefit of $264 068.10 and a total annualized financial benefit of $482 997.60 in recurring payments to patients. The calculated 2-year total financial benefit (total lump sum benefits + total annualized benefits  × 2) is thus $1.2 million in direct financial benefits to the cohort. All legal cases and outcomes were tracked until they were closed, with a median (IQR) time to closure of 66.5 (18.0-162.8) days, although the time to closure ranged from 1 day (eg, same day reinstatement of denied Medicaid benefits) to cases still active after 2 years (eg, denied disability benefits).

**Table 5.** Financial Benefits by Benefit Type Among Resolved Cases

Type	Cases, No. (%) (n = 134)	Mean benefit, $^a^	Total benefit, $
Monthly benefit			
SNAP	37 (27.6)	303.70	11 237.00
Medicaid or Medicare	11 (8.2)	606.73	6674.00
SSI or SSDI	18 (13.4)	939.78	16 916.01
TANF	3 (2.2)	614.67	1844.00
Other^b^	8 (6.0)	447.35	3578.79
Total	77 (57.5)	2912.22	40 249.80
Annualized total	NA	34 946.68	482 997.60
Lump sum benefit			
SNAP	16 (11.9)	337.81	5405.00
Medicaid or Medicare	4 (3.0)	2421.92	9687.66
SSI or SSDI	14 (10.4)	7356.15	102 986.12
TANF	4 (3.0)	1616.00	6464.00
Other^b^	6 (4.5)	997.57	5985.39
Rental debt dismissal	1 (0.7)	19 652.99	19 652.99
Crime Victims Compensation	12 (9.0)	9490.58	113 886.94
Total	57 (42.5)	41 873.01	264 068.10
Total financial benefit^c^	NA	NA	1 230 063.30

Abbreviations: NA, not applicable; SNAP, Supplemental Nutrition Assistance Program; SSDI, Social Security Disability Insurance; SSI, Supplemental Security Income; TANF, Temporary Assistance for Needy Families.

^a^ Calculated by dividing the total benefit for each benefit type by the number of benefits procured for each benefit type.

^b^ Other included benefits related to unemployment compensation, public utilities, housing, AABD cash assistance, and overpayments and waivers.

^c^ Estimated by multiplying the annualized total monthly benefit by the 2-year study period and adding the total lump sum benefit during the study period.

## Discussion

In this study of patients affected by violent injury in Chicago, Illinois, nearly all (94.8%) who were screened reported at least 1 legal need, with especially high need for income support (89.3%). Almost 9 in 10 participants reported needing assistance with access to the public benefits economy, with 25.0% reporting barriers to access in the past. To our knowledge, this is one of the first studies to quantify exclusion from the public benefits economy that specifically focused on a cohort of patients affected by violent injury in neighborhoods with concentrated economic disadvantage.

Our findings extend prior work by examining access to public benefits in conjunction with neighborhood economic disadvantage. Patients with a positive screen for income needs received public benefits at similar rates across all levels of disadvantage but reported a higher need for assistance in neighborhoods with higher disadvantage. Findings suggest that patients in neighborhoods with higher disadvantage may have more difficulty accessing benefits compared with patients in neighborhoods with lower disadvantage, even when reporting similar needs. Possible reasons include lack of awareness of potential benefits, difficulty navigating the complex system, or denial based on administrative or discriminatory policies.^10,14,17^ We conceptualize inadequate access regardless of the reason as exclusion from the public benefits economy, with adverse consequences for health and risk of firearm injury. By addressing this exclusion, we attempt to reframe the narrative around community violence from a personal and interpersonal pathology to a societal one.

This work also suggests that MLP is one possible and pragmatic solution to address economic exclusion as a core structural disadvantage associated with violent injury. Our program had an 85.1% cooperation rate and 94.8% positive screening rate for HHLNs, indicating high levels of feasibility, acceptability, and appropriateness among admitted patients. These preliminary findings have supported more formal prospective evaluation of our program through a clinical trial now under way. Furthermore, our MLP yielded a 2-year calculated total of $1.2 million in direct financial benefits for community members. By fortifying the social safety net for some of the most marginalized patients—those directly impacted by violent injury—MLPs may facilitate an opportunity for broader communitywide benefit and reparation.

Public debates surrounding benefits policies have often focused on whether benefits disincentivize employment, resulting in more stringent criteria for access.^18,19^ Prior studies, however, have documented that stringent criteria are associated with increased poverty and worsening health, without clear evidence for increasing employment.^18,19^ Alternatively, access to income support is associated with higher health care access, lower health care expenditure, and better health.^14,20,21^ A 2022 review additionally documented that income support policies were associated with reductions in the risk of community violence.^22^ For individuals recovering from violent injuries, public benefits may be instrumental in facilitating better recovery while preventing financial toxicity—both of which are needed to prevent compounding cycles of poverty and violence—thus increasing potential for future social participation and employment.

### Limitations

This study has several limitations. First, while we document that many patients have difficulty accessing the public benefits economy, we do not have data on the potentially vast and complex reasons for limited access. However, this is the first study of our program, and future qualitative work is planned to understand the mechanisms that contribute to limited access. Second, these data reflect the experiences of patients recovering from violent injuries at a single academic medical center on the South Side of Chicago. Results are generalizable to similar urban areas with high rates of residential segregation and concentrated poverty.

Third, all financial benefits reported in this study are from direct legal representation for public benefits through our program. It is possible that some patients applied for benefits on their own after legal consultation, which would result in case closure without documentation of a benefit. Similarly, financial benefits obtained outside of our program (eg, via social work) or indirect benefits resulting from nonmonetary case types (eg, housing) are not reflected in the financial data. Our findings may thus underestimate the total financial benefits received. For example, criminal records expungement is not quantified as a financial benefit but is a valuable intervention that should be quantified in future economic impact studies (ie, social return on investment). Additionally, financial benefits data were only available for cases that were closed during the study period, with many cases ongoing. Alternatively, it is possible that in an experimental design, patients in a control group would have been able to obtain benefits without legal assistance. Future studies are needed to determine causality, as well as mechanisms for potential outcomes.

## Conclusions

In this cohort study of patients admitted for violent injury at a level 1 trauma center in Chicago who were screened for HHLNs, we found a high burden of legal needs, particularly for income support with public benefits. One in 4 patients reported a barrier to access in the past, and 9 in 10 reported needing assistance with access to public benefits. Patients in the highest quartile of neighborhood disadvantage reported a substantially higher need for assistance, despite poverty levels that typically correspond with eligibility for assistance programs. Our MLP closed 409 (58.9%) cases during the study period and recovered a calculated 2-year total of $1.2 million in financial benefits to community members. MLP may be one possible solution for addressing exclusion from the public benefits economy—a structural disadvantage that limits economic stability, health, and safety among historically marginalized people.

## Notes

Supplement 1. **eTable 1.** Public Benefits Characteristics of Recovery Legal Care Participants Stratified by ADI, Chicago, Illinois, November 16, 2022-November 11, 2024

Supplement 2.
Data Sharing Statement
