# Supplementary material for: Income Support Needs and Bedside Legal Assistance for Patients Recovering From Violent Injuries
Source: JAMA Netw Open. 2025 Oct 16;8(10):e2538044. doi: 10.1001/jamanetworkopen.2025.38044 (PMC12531874; doi:10.1001/jamanetworkopen.2025.38044)
Supplement: Supplement 1. — eTable 1. Public Benefits Characteristics of Recovery Legal Care Participants Stratified by ADI, Chicago, Illinois, November 16, 2022-November 11, 2024 [file jamanetwopen-e2538044-s001.pdf]

Supplemental Online Content

Tung EL, Pillai R, Sen-Gupta N, Nigro A, Cosey-Gay F, Stolback BC, Rogers SO, Zakrison TL. Income support needs and bedside legal assistance for patients recovering from violent injuries. *JAMA Netw Open.* 2025;8(10): e2538044. doi: 10.1001/jamanetworkopen.2025.38044

eTable 1. Public Benefits Characteristics of Recovery Legal Care Participants Stratified by ADI, Chicago, Illinois, November 16, 2022-November 11, 2024

This supplemental material has been provided by the authors to give readers additional information about their work.

© 2025 Tung EL. et al. *JAMA Network Open*.

eTable 1. Public Benefits Characteristics of Recovery Legal Care Participants Stratified by ADI, Chicago, Illinois, November 16, 2022-November 11, 2024

**Area Deprivation Index (ADI) Quartile** N=418[a]	Currently receives benefit n (%)	Desires or needs assistance with benefit n (%)
Lowest and medium ADI quartiles[b]	34 (72.3)	36 (76.6)
High ADI quartile	114 (65.9)	135 (78.0)
Highest ADI quartile	141 (71.2)	171 (86.4)
Lowest and medium ADI quartiles	34 (72.3)	36 (76.6)
High and highest ADI quartiles	255 (68.7)	306 (82.5)

[a]Sample includes the 418 participants without missing address data.
[b]Values were pooled due to small numbers.

© 2025 Tung EL. et al. *JAMA Network Open*.
