# Supplementary material for: Income Support Needs and Bedside Legal Assistance for Patients Recovering From Violent Injuries
Source: JAMA Netw Open. 2025 Oct 16;8(10):e2538044. doi: 10.1001/jamanetworkopen.2025.38044 (PMC12531874; doi:10.1001/jamanetworkopen.2025.38044)
Supplement: Supplement 2. — Data Sharing Statement [file jamanetwopen-e2538044-s002.pdf]

Data Sharing Statement

Tung. Income Support Needs and Bedside Legal Assistance for Patients Recovering From Violent Injuries. *JAMA Netw Open*. Published October 16, 2025. doi:10.1001/jamanetworkopen.2025.38044

Data
Data available: No

Additional Information
Explanation for why data not available: Data are confidential with concern for identification.
